# Neurotoxic mechanisms of cadmium in neurodegenerative diseases

**DOI:** 10.3389/fcell.2026.1899381

**Published:** 2026-08-24

**Authors:** Zihao Zhang, Yutao Lu, Jiawei Yang, Meiren Li, Mingliu Yang, Yanhao Xu, Muhammad Saad Ullah, Qing Wan, Bing Bao, Wenmin Yu, Xiaoqun Liu

**Affiliations:** 1 School of Clinical Medicine, Jiujiang University, Jiujiang, Jiangxi, China; 2 Department of Neurology, Jiujiang Clinical Precision Medicine Research Center, Jiujiang, Jiangxi, China; 3 Department of Neurology, Affiliated Hospital of Jiujiang University, Jiujiang, China; 4 Department of Pharmacology, Yunnan Vocational College of Agriculture, Kunming, Yunnan, China; 5 Faculty of International Studies, Jiujiang University, Jiujiang, Jiangxi, China; 6 Center of Clinical Laboratory Medicine, Zhongda Hospital, Southeast University, Nanjing, China; 7 The School of Basic Medical Science, Jiujiang University, Jiujiang, Jiangxi, China; 8 Jiangxi Provincial Key Laboratory of Cell Precision Therapy, Jiujiang University, Jiujiang, Jiangxi, China

**Keywords:** cadmium, neurotoxicity, ferroptosis, neurodegenerative diseases, oxidative stress, inflammation

## Abstract

Cadmium (Cd) is a highly toxic, bioaccumulative heavy metal increasingly implicated in the pathogenesis of neurodegenerative disorders. This review systemically characterizes the molecular mechanisms underlying Cd-induced neurotoxicity, with particular emphasis on oxidative stress-mediated pathways that initiate interconnected processes including ferroptosis, mitochondrial impairment, disruption of calcium homeostasis, and chronic neuroinflammation. Evidence indicates that Cd exerts both convergent and disease-specific effects in neurodegenerative conditions. In Alzheimer’s disease (AD), Cd exposure has been associated with enhanced amyloid-β (Aβ) deposition and increased tau hyperphosphorylation. In Parkinson’s disease (PD), Cd disrupts metabolic homeostasis via the gut–liver–brain axis and promotes aberrant conformational changes and aggregation of α-synuclein (α-Syn). Within the amyotrophic lateral sclerosis–frontotemporal dementia (ALS–FTD) spectrum, Cd contributes to TDP-43 proteinopathy and impairs nucleocytoplasmic transport mechanisms. Therapeutic strategies targeting Cd-induced neurotoxicity are also explored, including upstream approaches like metal chelation and downstream interventions aimed at restoring autophagic flux, modulating the neuroimmune microenvironment, and enhancing neuronal repair. Although emerging platforms such as brain organoids provide valuable mechanistic insights, translating findings from *in vitro* models to real-world chronic exposure scenarios remains a significant challenge. This review provides a comprehensive framework for the development of early-warning systems and precision-based interventions for Cd-related neurodegeneration.

## Introduction

Cadmium (Cd) is a pervasive environmental heavy metal with a pronounced capacity for bioaccumulation in living systems. Its toxicity is primarily driven by complex redox disequilibrium and disruption of cellular homeostasis (Balali-Mood et al., 2021). Owing to its extremely slow rate of excretion and a prolonged biological half-life of up to 30 years, Cd progressively accumulates in multiple tissues, including those of the nervous system (Charkiewicz et al., 2023). Major sources of human exposure to Cd include industrial emissions, agricultural fertilizers, and chronic tobacco use, all of which contribute to the widespread public health burden (Kim et al., 2023).

Within the central nervous system, Cd can traverse the blood–brain barrier (BBB) or access neural tissue via the olfactory pathway, thereby initiating neurodegenerative processes through a cascade of interconnected molecular events. The global burden of neurodegenerative disorders, including Alzheimer’s disease (AD), Parkinson’s disease (PD), and amyotrophic lateral sclerosis (ALS), has increased substantially in recent decades, underscoring the need to identify modifiable environmental risk factors (Erkkinen et al., 2018). Emerging evidence indicates that Cd ions disrupt amyloid-β (Aβ) homeostasis, facilitate the formation of neurotoxic ion channels (Notarachille et al., 2014), and promote the misfolding and aggregation of pathogenic proteins.

Given the ubiquity of Cd exposure, elucidating the shared molecular pathways underlying its neurotoxic effects across diverse neurodegenerative diseases is of critical importance for both mechanistic research and public health. Such insights may enable the identification of early biomarkers and the development of targeted therapeutic strategies for Cd-associated neurodegeneration.

## Core molecular mechanisms of cadmium neurotoxicity: common pathways across diseases

### Oxidative stress and the collapse of the antioxidant defense system

Although cadmium, as a non-redox-active metal, cannot directly drive Fenton-like reactions similar to iron or copper, it triggers oxidative stress indirectly by disrupting cellular metal homeostasis, which serves as the initiating stage of neurotoxicity (Branca et al., 2020). This collapse of redox homeostasis involves multiple interference effects of cadmium ions: on the one hand, due to the extremely high affinity of cadmium ions (Cd^2+^) for sulfhydryl groups (-SH), they extensively deplete intracellular reduced glutathione (GSH), thereby directly weakening the biochemical defense capacity of neurons to eliminate hydrogen peroxide (H_2_O_2_) (Valko et al., 2016); on the other hand, Cd^2+^ may competitively displace Zn^2+^ from the active center of Cu/Zn–superoxide dismutase (SOD) through structural mimicry.

This competitive ion displacement leads to changes in enzyme conformation and subsequent inactivation, blocking the normal conversion of superoxide anions at the source (Arruebarrena et al., 2023). The systemic disintegration of this defense system disrupts the dynamic balance of intracellular oxidation, leaving neurons exposed to persistent oxidative stress. As the activities of key antioxidant enzymes such as CAT and GPx are generally inhibited, uncontrolled reactive oxygen species (ROS) begin to indiscriminately attack the membranous structures of neurons. This initiates a cascade reaction, leading to a significant increase in the levels of malondialdehyde (MDA), a marker of lipid peroxidation, in brain tissue (Oviosun et al., 2025). As an end-product of lipid peroxidation, MDA directly mediates cytotoxicity by forming adducts with DNA and proteins. Furthermore, this loss of membrane lipid integrity does not occur in isolation; rather, it signifies that intracellular pro-oxidant markers can no longer be counteracted. When the GPx4-mediated reduction pathway for phospholipid hydroperoxides becomes decompensated, it establishes the necessary conditions to trigger metabolic ferroptosis (Gong et al., 2025).

This systemic failure of antioxidant defenses indicates toward concrete therapeutic entry points, notably restoration of GSH and reactivation of Cu/Zn–SOD. Several phytochemicals and nutritional interventions that act on these oxidative stress nodes, including resveratrol, celastrol, quercetin, and selenite, are considered in the *Therapeutic Strategies* section (Table 1), where their capacity to rebalance redox homeostasis and curb downstream lipid peroxidation is examined in detail.

**TABLE 1:** Summary of major intervention strategies for cadmium-induced neurotoxicity, including detoxification, drug repurposing, phytochemicals, nutritional interventions, and novel molecular designs.

Category	Intervention	Targets and mechanisms	Model and target region	Key outcomes	Reference
Systemic detoxification	EDTA	Directly binds to systemic toxic metal ions	Human clinical cohorts	Reduces systemic toxic burden; improves degenerative symptoms	Fulgenzi et al. (2020) and Ferrero (2022)
	Chitosan-based polymer	Captures free metals in the gastrointestinal tract	Rat systemic exposure model	Blocks entero-blood absorption; prevents trace element loss	How et al. (2023)
Drug repurposing	Metformin	Targets the ROS–PP5/AMPK–JNK axis to inhibit apoptosis	Neurons; auditory SGNs	Blocks sensory nerve degeneration; prevents apoptosis	Li et al. (2022) and Chen et al. (2020)
	Linagliptin	Activates the SIRT1/Nrf2 axis to restart cellular autophagy	Rat neurotoxicity model	Clears toxic proteins; reverses memory deficits	Arab et al. (2023a)
	Quetiapine	Upregulates Keap1/Nrf2; downregulates NF-κB and NLRP3	*In vivo* animals/*in vitro* cells	Blocks the inflammatory cascade; downregulates degenerative markers	Althagafy et al. (2024)
Phytochemicals	Resveratrol	Inhibits abnormal mTORC1/mTORC2; activates PP2A/PP5 and inactivates Erk1/2 and JNK	*In vitro* neuronal apoptosis model	Restarts autophagic recycling; highly improves the survival rate	Liu et al. (2022) and Liu et al. (2015)
	Celastrol	Upregulation of PTEN inhibits Akt/mTOR; targeting NOX2 suppresses ROS and restores the PP5–JNK balance	Neuronal apoptosis model	Remodels the kinase network; blocks apoptosis at the source	Xu et al. (2017) and Chen et al. (2014)
	Quercetin	Regulates NMDA-R excitability; activates the PI3K/AKT–Nrf2 axis	Rat hippocampus	Protects synaptic integrity; improves cognition	Srivastava et al. (2023)
	Curcumin	Neutralizes excess ROS and suppresses inflammatory proteins in the target brain region	Mouse PFC	Alleviates emotional disorders and anxiety-like behaviors	Namgyal et al. (2021)
	Puerarin	Stimulates neurons to actively pump accumulated cadmium ions out of the cell	Neuronal cytotoxicity model	Directly reduces intracellular toxic concentration	Wen et al. (2021)
	Vanillylacetone	Potently upregulates Nrf2 expression and its downstream targets SOD and GSH and inhibits Bax expression	Rat hippocampus	Reverses structural damage; restores spatial memory	AL-Hashem et al. (2024)
Nutritional interventions	Soybean-based diet	Competitively displaces toxic cadmium ions	Rat cerebellum	Maintains essential metal balance; avoids morphological changes	Martin Molinero et al. (2023)
	Selenite	Upregulates thioredoxin reductase 1 (TrxR1)	Human SH-SY5Y cells	Reverse necrosis via endogenous biochemical defense	Wang et al. (2023b)
	L-Theanine	Prevents the abnormal aggregation and excessive phosphorylation of tau proteins	Early degeneration model	Defends against AD-like microtubule structural collapse	Ben et al. (2016)
	Gallic + ascorbic acids	Neutralizes free radicals	Rat widespread brain lesion model	Prevents widespread structural damage and DNA breaks	Adebiyi et al. (2022)
Emerging design	MTDLs: 7-aminophenanthridin-6-one	Metal chelation; inhibits amyloid aggregation	AD-like complex toxicity model	Extracts pathogenic metals; disrupts toxic protein plaques	Moyano et al. (2022)

### Ferroptosis: lipid peroxidation and dysregulation of iron metabolism

**FIGURE 1:**
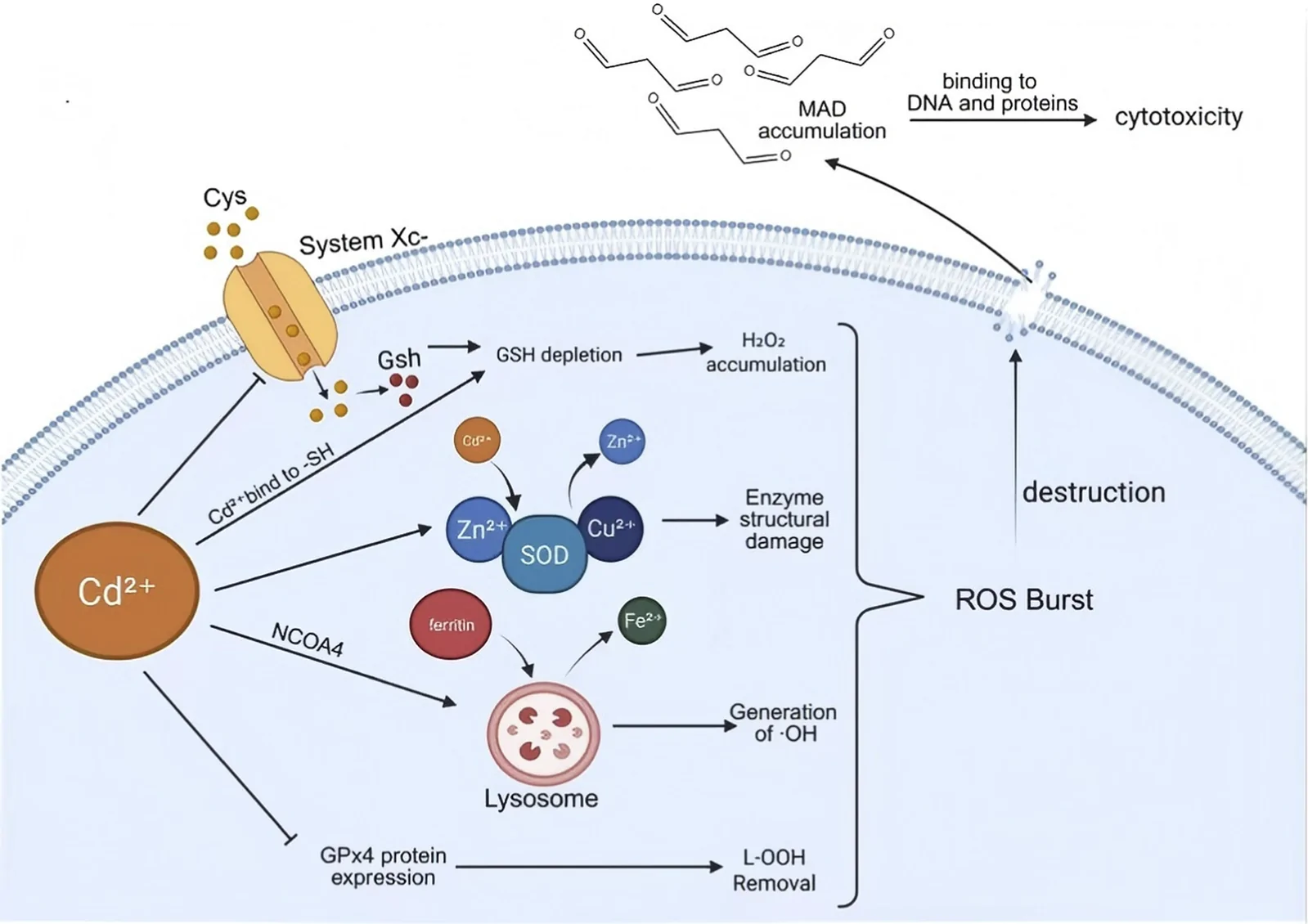
Mechanism of Cd^2+^-induced neuronal ferroptosis. The diagram illustrates the systemic failure of antioxidant defenses, including GSH depletion and SOD inactivation, alongside the activation of the System Xc^−^/GPx4 axis and NCOA4-mediated ferritinophagy, resulting in lipid peroxidation and loss of neuronal membrane integrity.

As illustrated in Figure 1, when oxidative damage to membrane lipids exceeds the cellular repair capacity, neurons shift from adaptive stress responses to an irreversible form of cell death known as ferroptosis. This process is predominantly driven by Cd, which disrupts the System Xc^−^/glutathione peroxidase 4 (GPx4) axis and perturbs intracellular iron homeostasis. Inhibition of System Xc^−^ by Cd limits cystine uptake, thereby depleting substrates required for glutathione biosynthesis. Concurrently, Cd downregulates GPx4 expression, resulting in the functional inactivation of this key lipid peroxide-scavenging enzyme (Zhang X. et al., 2025). Several converging mechanisms drive this inactivation. Because GPx4 is a selenoprotein with the catalytic activity depending on an active-site selenocysteine residue, Cd represses GPx4 at the transcriptional level (Messaoudi et al., 2010). *In vivo*, this suppression is dose- and time-dependent: Cd administration reduces Gpx4a expression by 13%–40% and that of Gpx4b by 18%–37% (Adiele et al., 2012). Cd also disrupts selenium homeostasis: coordinate loss of selenoprotein P and GPx4 tracks with depleted tissue selenium, a deficit that selenium supplementation can substantially reverse (Golin et al., 2025). Finally, Cd-derived ROS further impair GPx4 through deleterious post-translational modifications (Hong et al., 2022). Together, these mechanisms decisively skew the cellular redox balance, creating a microenvironment highly permissive to ferroptosis.

Beyond the System Xc^−^/GPx4 pathway, Cd also induces ferritinophagy via nuclear receptor coactivator 4 (NCOA4). This selective autophagic process promotes lysosomal degradation of ferritin, leading to the release of labile ferrous iron (Fe^2+^) (Wang D. et al., 2024), which fuels the Fenton reaction. The resultant generation of highly reactive hydroxyl radicals (•OH) further amplifies lipid peroxidation (Hao et al., 2022). The signaling that drives NCOA4 activation is tightly coupled to Cd-induced oxidative stress. Accumulating intracellular ROS trigger autophagic flux, simultaneously activating the AMPK–ULK1 axis and suppressing mTORC1. ULK1 then phosphorylates NCOA4 directly, promoting its interaction with ferritin heavy chain 1 (FTH1) and targeting the complex for lysosomal clearance (Gu et al., 2026; Jia et al., 2025). Although the precise mechanistic details are still being elucidated, current models hold that Cd hijacks canonical autophagic pathways to mediate ferritinophagy.

Thus, therapeutic strategies targeting NCOA4-mediated ferritinophagy or restoring GPx4 activity may offer promising avenues for mitigating Cd-associated neurodegeneration. As detailed in the *Therapeutic Strategies* section (Table 1), phytochemicals such as resveratrol and celastrol inhibit ferroptosis by preserving GPx4 and suppressing ROS, whereas selenite directly supports GPx4 biosynthesis, although the translational feasibility of these approaches remains to be established.

### Mitochondrial dysfunction and energy crisis

When Cd enters the neurons, the mitochondria represent primary targets of Cd toxicity. When antioxidant defense systems are overwhelmed, mitochondrial oxidative damage is markedly exacerbated. Cd disrupts the electron transport chain (ETC) within the inner mitochondrial membrane by binding to respiratory complexes and impairing electron transfer (Peana et al., 2022), resulting in pronounced electron leakage and excessive generation of mitochondrial reactive oxygen species (mtROS) (Zhao et al., 2020).

Compromise of ETC function leads to a rapid dissipation of the mitochondrial membrane potential (ΔΨm), thereby abolishing the electrochemical gradient required for ATP synthesis and precipitating a severe cellular energy deficit. In response to this stress, mitochondrial permeability transition pores (mPTPs) undergo nonspecific opening, a phenomenon termed mitochondrial permeability transition (Kamitsuka et al., 2023). This event promotes outer mitochondrial membrane permeabilization and facilitates the release of pro-apoptotic and immunostimulatory factors, including cytochrome c and mitochondrial DNA (mtDNA), into the cytosol. Cytosolic release of mtDNA has been identified as a critical early event in the activation of the cyclic GMP–AMP synthase–stimulator of interferon gene (cGAS–STING) signaling pathway (Zhang C.-Y. et al., 2024). Mechanistically, mtDNA extrusion into the cytosol is not a passive consequence of organellar rupture but a tightly regulated process: oligomerization of voltage-dependent anion channels (VDACs) and assembly of BAX/BAK macropores in the outer mitochondrial membrane follow mPTP opening. Once in the cytosol, mtDNA acts as a potent damage-associated molecular pattern (DAMP) that is rapidly detected by cGAS, which catalyzes synthesis of the second messenger 2′3′-cGAMP and thereby activates STING. Activated STING translocates from the endoplasmic reticulum to the Golgi apparatus, where it scaffolds the recruitment of TANK-binding kinase 1 (TBK1); the resulting complex phosphorylates interferon regulatory factor 3 (IRF3), driving the transcription of type I interferons and proinflammatory cytokines (Giordano et al., 2025; Li et al., 2024; Bahat et al., 2025). This cGAS–STING cascade, thus, forms a direct molecular link between Cd-induced mitochondrial collapse and the onset of innate neuroinflammation.

### Calcium dyshomeostasis and signaling abnormalities

In Cd-induced neurotoxicity, disruption of intracellular calcium homeostasis represents a key pathological mechanism. Cd can enter cells by exploiting its physicochemical similarity to calcium ions (Ca^2+^), a phenomenon often described as ionic mimicry. This property enables Cd to interfere with normal calcium transport and signaling, thereby perturbing tightly regulated intracellular Ca^2+^ dynamics (Matsushita and Xia, 2024).

Evidence indicates that Cd impairs the activity of Ca^2+^-ATPases localized on the plasma membrane and intracellular organelles, leading to reduced efficiency of active calcium extrusion and sequestration (Wang and Du, 2013; Yuan et al., 2013). Consequently, the maintenance of calcium gradients across cellular and organellar membranes is compromised. Rather than causing a simple ionic imbalance, Cd induces a multifactorial disruption of calcium signaling networks.

The resulting intracellular calcium overload activates calcium-dependent proteases and other downstream effectors, ultimately contributing to neuronal injury and cell death through complex stress signaling cascades (Xu et al., 2021). Cd-induced cytosolic calcium overload also drives pathological opening of the mitochondrial permeability transition pore (mPTP). Ca^2+^ entering the matrix through the mitochondrial calcium uniporter (MCU) acts together with local oxidative stress to promote CypD-dependent mPTP sensitization; in parallel, Ca^2+^-activated calpains cleave OPA1, compromising inner membrane integrity and lowering the threshold for pore assembly. The resulting mPTP opening collapses the mitochondrial membrane potential (ΔΨm) and releases pro-apoptotic factors into the cytosol, establishing a self-amplifying cycle of calcium dyshomeostasis and irreversible mitochondrial degeneration (Yuan et al., 2013; Romanova et al., 2025; Garcia et al., 2025).

### Ignition and maintenance of neuroinflammation

Beyond its direct neurotoxic effects, Cd exposure establishes an uncontrolled and persistent inflammatory network in the brain. Microglia are particularly susceptible to Cd and rapidly adopt a proinflammatory phenotype via activation of the MAPK/NF-κB signaling pathway, resulting in the robust secretion of proinflammatory cytokines such as tumor necrosis factor-α (TNF-α) (Huat et al., 2019). This inflammatory response does not occur in isolation; concomitant activation of reactive astrogliosis further disrupts neuronal metabolic support, thereby accelerating neurodegeneration (Ma et al., 2025).

Hyperactivated glial cells directly compromise neural circuit integrity. Cd exposure induces aberrant complement-mediated phagocytic activity in microglia, leading to inappropriate elimination of functional synaptic elements and a reduction in synaptic protein levels (Li et al., 2025). In parallel, perturbations in iron metabolism and STEAP3–SLC39A8-mediated apoptotic signaling contribute to the release of DAMPs, thereby exacerbating immune dysregulation (Sun et al., 2025).

At the molecular level, Cd promotes the accumulation of mtROS through inhibition of sirtuin 3 (SIRT3) and enhanced acetylation of superoxide dismutase 2 (SOD2), resulting in activation of the NLRP3 inflammasome. The subsequent release of interleukin-1β (IL-1β) reinforces NF-κB signaling, thereby establishing a deleterious positive feedback loop that sustains neuroinflammation even after cessation of Cd exposure (Cai et al., 2021; Wang D. et al., 2023).

Accordingly, disruption of NLRP3 inflammasome-driven glial activation and reprogramming of the neuroimmune microenvironment represent promising therapeutic strategies, shifting the focus from conventional neuroprotection toward immune modulation in Cd-associated neurodegeneration. These strategies are examined further in the *Therapeutic Strategies* section (Table 1), where quetiapine and curcumin are noted for their ability to suppress NLRP3 activation and dampen neuroinflammation, although their clinical translation is constrained by limited target specificity and poor BBB penetration.

### Synaptic structural and functional dysfunction

Neurotoxicity associated with Cd is closely linked to structural and functional disruption of synapses. These deleterious effects initially manifest as impaired signal transmission at the presynaptic terminal. Owing to its physicochemical similarity to zinc ions (Zn^2+^), Cd perturbs Zn^2+^ homeostasis and associated transport systems, thereby disrupting synaptic vesicle function.

Evidence indicates that Cd competes with both calcium and zinc for critical binding sites, leading to dysregulated release of inhibitory neurotransmitters, including γ-aminobutyric acid (GABA) and glycine (Yu et al., 2021). This disruption compromises synaptic fidelity and impairs effective neuronal communication. Furthermore, ionic imbalance not only reduces synaptic transmission efficiency but also induces neuronal stress by disturbing intracellular cation homeostasis (Cirovic et al., 2024).

The neurotoxic effects of Cd extend beyond perturbation of ionic fluxes to encompass profound disruption of enzymatic systems essential for neuronal signaling. Evidence indicates that Cd exposure significantly inhibits acetylcholinesterase (AChE) activity in brain tissue, resulting in the accumulation of acetylcholine within the synaptic cleft and consequent cholinergic dysfunction. Moreover, Cd dysregulates Na^+^/K^+^-ATPase activity, thereby disturbing intracellular ionic gradients, altering resting membrane potential, and impairing the fidelity of action potential propagation (Ruczaj et al., 2024). Experimental studies further suggest that Cd interferes with enzymes involved in energy metabolism, including nucleoside triphosphate diphosphohydrolases (NTPDases), leading to compromised ATP availability required for synaptic function (Senger et al., 2006).

Collectively, this widespread enzymatic dysregulation undermines the biochemical foundation of neuronal communication by disrupting both energy homeostasis and signal transduction pathways, thereby providing a mechanistic basis for impaired synaptic plasticity and cognitive dysfunction.

Several agents that preserve synaptic integrity, such as tetrahydroxystilbene glucoside (TSG), quercetin, and camphor, are reviewed in the *Therapeutic Strategies* section (Table 1), where their contrasting mechanisms (structural preservation through BDNF/TrkB versus functional enhancement via NMDA-receptor modulation and AChE inhibition) are compared.

As synaptic dysfunction and biochemical perturbations accumulate beyond a critical threshold, apoptotic pathways are activated, representing a pivotal stage at which Cd irreversibly compromises neuronal structural integrity. Evidence indicates that excessive ROS generated during mitochondrial dysfunction activate key signaling cascades, including the Akt/mTOR and mitogen-activated protein kinase (MAPK) pathways (encompassing JNK, p38, and ERK1/2). These events disrupt the balance between anti-apoptotic B-cell lymphoma 2 (Bcl-2) and pro-apoptotic Bcl-2-associated X protein (Bax), thereby initiating the caspase-3-dependent apoptotic cascade (Yan, 2016). Mitochondrial oxidative stress plays a central role in amplifying these apoptotic signals. Studies have demonstrated that mtROS induced by Cd exposure promotes inactivation of protein phosphatase 2A (PP2A), thereby relieving inhibitory control over MAPK signaling. This results in sustained pathway phosphorylation and potentiation of pro-apoptotic signaling, ultimately leading to irreversible neuronal damage (Xu et al., 2016). Collectively, the convergence of these signaling networks, in conjunction with glial cell-mediated inflammatory responses, constitutes the molecular framework underlying Cd-induced neuronal apoptosis.

**FIGURE 2:**
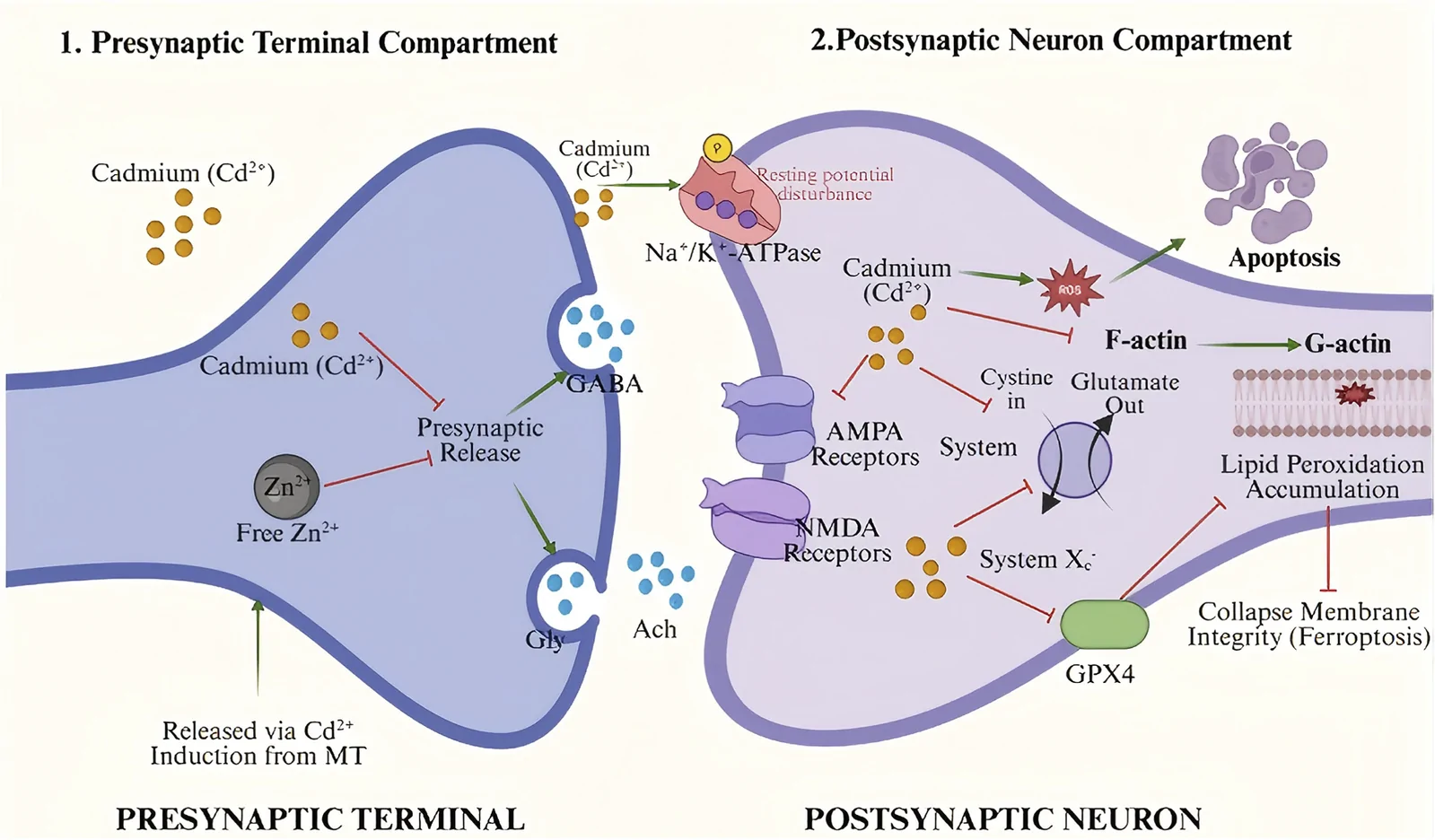
Schematic overview of cadmium (Cd^2+^)-induced neurotoxicity at the presynaptic and postsynaptic compartments.

The mechanisms described in this study do not operate in isolation; rather, they constitute an interconnected network through which Cd exacerbates neurodegenerative processes. Achieving a comprehensive understanding of these complex interactions remains a significant challenge for the development of multifaceted therapeutic strategies targeting Cd accumulation. The subsequent sections delineate the associations between Cd exposure and specific neurodegenerative diseases, thereby providing a framework to guide future experimental investigations. These interacting pathways and the resulting structural deterioration are summarized schematically in Figure 2.

## Association of cadmium with specific neurodegenerative diseases

### Epidemiological evidence: real-world data on environmental exposure and cognitive decline

To elucidate the relationship between Cd exposure and neurotoxicity, a growing body of epidemiological studies have established a convergent line of evidence across diverse populations. Early investigations focusing on adults aged 20–59 years highlight the cumulative impact of chronic Cd exposure. Analyses of urinary Cd concentrations revealed a significant inverse association between environmental Cd exposure and performance on neurocognitive assessments, an effect that persisted after adjustment for potential confounders. These findings suggest that Cd-related neurotoxicity may contribute to early-stage cognitive decline (Ciesielski et al., 2013).

The magnitude of these effects appears more pronounced in older populations. Multivariate analyses of individuals aged ≥60 years identified elevated blood Cd levels as an independent risk factor for impaired cognitive function. Notably, a significant interaction has been observed between dietary omega-6 polyunsaturated fatty acids and Cd exposure, whereby high-fat dietary patterns exacerbate Cd-induced neurotoxicity, underscoring the modulatory role of nutritional factors in older adults (Huang and Ren, 2022). Importantly, even after accounting for protective trace elements such as serum iron and manganese, generalized linear models demonstrate that blood Cd levels remain significantly associated with reduced performance on the digit symbol substitution test (DSST), indicating a primary impact on processing speed and white matter integrity (Lu et al., 2023). To address the complexity of mixed-metal exposures, Bayesian kernel machine regression (BKMR) models have been employed to segregate individual and combined effects. These analyses identified Cd as the predominant contributor to deficits in immediate recall within the context of multi-metal exposure (including lead, mercury, and selenium), implicating hippocampus-dependent memory functions (Fu et al., 2024).

Much of the foundational population data on cadmium toxicity come from Western NHANES cohorts, which have linked dietary and tobacco smoke exposure to neurological outcomes such as Parkinson’s disease (Tu et al., 2024). Understanding the global burden, however, requires evidence from large-scale Asian studies. In China, industrial and agricultural activities have heavily contaminated farmland, and more than 7% of the country’s soils are reportedly severely polluted with cadmium (Chu et al., 2026). Locally grown rice is, therefore, a major exposure route, and national dietary assessments identify this staple as the single largest contributor to cadmium intake in the general Chinese population (S et al., 2017). This dietary burden is often compounded by regional industrial emissions and tobacco use: large adult cohorts show markedly elevated blood cadmium in smokers and in residents of industrialized urban areas (Nie et al., 2016). Taken together, these cohorts show how contaminated soil, staple diets, industrial activity, and smoking combine into a cumulative exposure profile that provides a crucial real-world context for assessing neurodegenerative risk worldwide.

Furthermore, advances in artificial intelligence are facilitating earlier detection of Cd-associated neurotoxicity. Machine learning approaches, including random forest and extreme gradient boosting (XGBoost), have identified heavy metal exposure as a key predictive variable for cognitive decline, demonstrating predictive performance that surpasses that of certain conventional biomarkers. These findings provide a foundation for the development of early warning systems and highlight novel avenues for the prevention of neurodegenerative disorders (Nabavi et al., 2024).

Collectively, although epidemiological evidence underscores the widespread impact of Cd on cognitive function, the underlying molecular mechanisms may differ across neurodegenerative diseases, reflecting variability in the affected brain regions and disease-specific pathogenic pathways.

### Alzheimer’s disease

Accumulating evidence suggests that environmental Cd exposure may act as a contributory factor in the progression from cognitive impairment to AD. Epidemiological data demonstrate an inverse association between blood Cd levels and cognitive performance, particularly among individuals with mild cognitive impairment (MCI) (Urbano et al., 2024).

Importantly, the impact of Cd exposure may not be confined to aging populations. Experimental studies indicate that exposure during critical developmental windows, including gestation and lactation, can induce persistent deficits in synaptic plasticity that manifest in adulthood. Mechanistically, these effects have been linked to inhibition of the phospholipase Cβ4 (PLCβ4)/brain-derived neurotrophic factor (BDNF) signaling pathway in offspring, ultimately contributing to long-term cognitive dysfunction (Wang Y. et al., 2024).

Recent investigations have increasingly focused on the gut–liver–brain axis as a critical mediator of Cd-induced neurotoxicity. In a genetic susceptibility model employing apolipoprotein E4 (ApoE4) transgenic mice, early-life Cd exposure was shown to significantly alter gut microbiota composition, subsequently impairing hepatic metabolic detoxification capacity. These findings challenge the conventional view that Cd exerts direct hepatotoxic effects on detoxification pathways, instead highlighting an indirect, microbiota-mediated mechanism of systemic toxicity (Zhang et al., 2021). Given the pivotal role of the liver in the peripheral clearance of Aβ, hepatic dysfunction may facilitate the accumulation of circulating Aβ, which can subsequently access the central nervous system via compromised BBB transport mechanisms. This process may contribute to the initiation and progression of AD, a process that is closely related to gender (Wang et al., 2022).

This mechanistic framework, however, rests almost entirely on ApoE4 transgenic mice, a model that captures a single genetic risk allele and does not reflect the polygenic, heterogeneous nature of sporadic AD. Direct clinical evidence for Cd-induced gut microbiota dysbiosis, impaired hepatic bile acid metabolism, or reduced TUDCA synthesis in humans is notably lacking. The translational limits of these animal-derived conclusions, particularly interspecies differences in bile acid enterohepatic circulation, gut microbial composition, and Cd toxicokinetics, also remain poorly defined. Until cross-species comparisons and prospective human cohorts are available, the causal role of the gut–liver–brain axis in Cd-associated AD should be interpreted with caution.

Cd accelerates the two principal pathological hallmarks of AD: Aβ accumulation and tau hyperphosphorylation. Although the precise interplay between these processes in the context of Cd-induced neurotoxicity remains to be fully elucidated, the Aβ pathway contributes to AD pathogenesis through two primary mechanisms.

First, Cd enhances the amyloidogenic processing of the amyloid precursor protein (APP) by upregulating the expressions of β-site APP-cleaving enzyme 1 (BACE1) and presenilin-1 (PS1), thereby promoting the generation of the neurotoxic Aβ_1_–_42_ isoform (Ali et al., 2021). Second, Cd disrupts intracellular calcium and zinc homeostasis, creating a microenvironment that facilitates the aggregation of Aβ monomers into toxic oligomeric species, thereby amplifying neurotoxicity (Corona et al., 2011).

This neurotoxic impact extends to the tau protein network. Cd-induced impairment of the M1 muscarinic acetylcholine receptor (M1 mAChR) is considered a critical early event that disrupts downstream neuroprotective signaling. This perturbation leads to dysregulation of glycogen synthase kinase-3β (GSK-3β), promoting aberrant tau phosphorylation at key residues, including Ser202 and Thr205. Consequently, tau dissociates from microtubules and undergoes pathological aggregation. These events culminate in the formation of neurofibrillary tangles (NFTs), which accelerate neurodegenerative processes within neuronal cell bodies (Del Pino et al., 2016).

Concurrently, Cd-induced alterations in thyroid hormone homeostasis, together with sustained oxidative stress, contribute to a proinflammatory molecular environment that further exacerbates protein misfolding, enhances NFT formation, and promotes neuronal loss and cognitive decline (Kim et al., 2018).

Although these mechanisms appear well defined, current mechanistic models are largely derived from studies on high-dose exposure. Determining whether chronic low-dose Cd exposure can elicit comparable kinase activation and downstream pathological cascades remains a critical challenge for future research.

Cd-induced neurotoxicity fundamentally reflects the disruption of intracellular proteostasis networks. Within the autophagy–lysosome pathway, Cd promotes aberrant lysine 31 (K31) succinylation of the small GTPase Ras-related protein Rab-7a (Rab7a) by inhibiting the expression of desuccinylase sirtuin 5 (SIRT5). This pathological post-translational modification impairs autophagosome–lysosome fusion, thereby compromising the clearance of neurotoxic aggregates, including Aβ (Deng et al., 2024).

Concurrently, Cd disrupts endoplasmic reticulum-associated degradation (ERAD) by accelerating the ubiquitination and proteasomal degradation of the neuroprotective protein sigma-1 receptor (SigmaR1) via the SEL1L–HRD1 complex. This leads to loss of critical neuroprotective functions (Qian et al., 2024).

Collectively, these convergent defects drive progressive neuronal dysfunction and cell death, thereby accelerating neurodegenerative processes associated with AD.

Disruption of proteostasis is not restricted to AD-associated proteins. α-Synuclein (α-Syn), a protein highly dependent on autophagic clearance, may also accumulate in Cd-exposed neural tissue, thereby contributing to broader neurodegenerative pathology, including AD-related processes (Park et al., 2025).

However, much of the existing literature remains grounded in reductionist frameworks that emphasize single-target, single-outcome paradigms. Given that Cd induces widespread impairment of autophagic flux alongside dysregulation of multiple protein networks, therapeutic strategies targeting isolated pathways are unlikely to achieve meaningful clinical efficacy.

These observations underscore the necessity of shifting toward more multifaceted approaches that focus on network remodeling.

### Parkinson’s disease

Epidemiological evidence indicates that environmental Cd exposure is significantly associated with an increased risk of PD. Recent analyses derived from the National Health and Nutrition Examination Survey (NHANES) cross-sectional dataset demonstrate a positive association between urinary Cd concentrations and PD prevalence, supporting Cd as an independent risk factor for the disease (Lv et al., 2025).

Analogous to AD pathogenesis, Cd-associated PD progression involves dysregulation of the gut–liver–brain axis. Cd-induced alterations in gut microbiota composition markedly reduce the production of neuroprotective metabolites, particularly short-chain fatty acids (SCFAs), thereby weakening central anti-inflammatory defenses. This impairment in neuroimmune regulation is initiated peripherally at the level of the gut (Kollaparampil Kishanchand et al., 2025).

Concurrently, Cd-mediated perturbations in hepatic bile acid metabolism, most notably reduced synthesis of tauroursodeoxycholic acid (TUDCA), a bile acid with established neuroprotective and mitochondrial-stabilizing properties, result in diminished support for mitochondrial integrity in dopaminergic neurons of the substantia nigra (Hurley et al., 2022).

This systemic metabolic dysregulation, characterized by depletion of beneficial microbial metabolites and bile acid imbalance, facilitates the misfolding and propagation of α-Syn within the enteric nervous system, while simultaneously exacerbating oxidative stress in the central nervous system. Collectively, aberrant integration of peripheral metabolic signals into central neurodegenerative pathways constitutes a key mechanistic framework underlying Cd-associated PD pathogenesis (Yan et al., 2023).

Beyond these mechanisms, Cd can directly induce misfolding and aggregation of α-Syn, thereby accelerating PD progression. On the one hand, Cd ions can interact with α-Syn and act as catalytic cofactors that promote its fibrillation (Lorentzon et al., 2021). On the other hand, α-Syn pathology contributes to a deleterious feed-forward cycle of Cd accumulation, whereby neurons with elevated α-Syn expression exhibit increased Cd uptake, leading to enhanced ROS generation and activation of the intrinsic apoptotic pathway via caspase-9 and caspase-3 (Chong et al., 2018).

With both structural integrity and metabolic homeostasis compromised, neuronal injury perpetuates a self-amplifying cycle of degeneration. In parallel, Cd disrupts BBB integrity by downregulating the tight junction protein zonula occludens-1 (ZO-1) and inducing sustained activation of pannexin-1 channels. This results in pathological ATP efflux and exacerbation of neuroinflammatory signaling (Branca et al., 2019).

Neuroinflammation plays a central role in the pathogenesis of PD, and its exacerbation contributes to both disease initiation and progression. These inflammatory processes interact with metabolic disturbances originating from the gut–liver axis, resulting in progressive degeneration of the substantia nigra. This degeneration reflects a convergence of persistent peripheral inflammatory signaling and intracellular proteotoxic stress, ultimately driving an irreversible neurodegenerative state.

Cd-related neurodegeneration resembling PD fundamentally arises from dysregulation of the interplay between mitochondrial maintenance mechanisms and protein degradation systems. Similar to Cd-induced impairment of autophagy via SIRT5 in AD, Cd disrupts mitophagy in PD models, leading to the accumulation of dysfunctional mitochondria (Wen et al., 2022). This, in turn, promotes ferroptosis and drives dopaminergic neurons toward irreversible degeneration (Sahoo and Sharma, 2023).

The progressive and selective loss of dopaminergic neurons in the substantia nigra pars compacta constitutes the core pathological substrate underlying the depletion of striatal dopamine levels, thereby directly contributing to the onset and progression of PD (Liang et al., 2025). Given the multifactorial nature of these pathological processes, therapeutic strategies targeting a single pathway are often insufficient.

Accordingly, future approaches for managing environmentally induced PD should move beyond a narrow focus on antioxidant therapy toward integrated strategies that encompass modulation of the gut microbiota, regulation of hepatic metabolism, and restoration of neuronal proteostasis.

### Amyotrophic lateral sclerosis: a mechanistic model for cognitive degeneration

The distinctions between ALS and frontotemporal dementia (FTD) are increasingly being reconsidered due to their overlapping clinical features and the shared pathology of TDP-43 proteinopathy. Approximately 50% of individuals with ALS exhibit cognitive or behavioral impairments, and up to 15% meet the diagnostic criteria for FTD. These observations indicate that Cd exposure may contribute to subclinical cognitive decline through its deleterious effects on cortical neurons (Michielsen et al., 2025; Kirola et al., 2022).

Supporting this, prospective cohort studies have demonstrated a positive association between elevated Cd levels in the blood and both the risk of developing ALS and the rate of neurological deterioration, providing epidemiological evidence for Cd as a contributing factor in neurodegeneration (Peters et al., 2021).

Cd-induced ALS-like neurodegeneration is primarily driven by disruptions in nucleocytoplasmic transport and aberrant immune activation. Cd exposure disrupts the integrity of the nuclear pore complex, thereby impairing nucleocytoplasmic transport and altering protein localization. This dysregulation promotes aberrant ubiquitination of the key pathological protein TDP-43, facilitating its cytoplasmic mislocalization (Tran and Lee, 2022).

This process not only accelerates neuronal degeneration but may also permit the release of mtDNA into the cytosol, thereby activating an intrinsic inflammatory response via the cGAS–STING pathway and directly contributing to the loss of sensitive cortical neurons (Marques et al., 2024).

In the context of disrupted cellular homeostasis involving oxidative stress, proteotoxicity, and neuroinflammation, most therapeutic strategies have predominantly targeted downstream apoptotic pathways while overlooking upstream pathogenic mechanisms (Genin and Paquis-Flucklinger, 2026; Kim et al., 2025). Future research should, therefore, emphasize the integrated role of impaired nucleocytoplasmic transport and aberrant immune signaling, with a focus on upstream molecular targets to more effectively mitigate the incidence and progression of Cd-related ALS.

## Frontotemporal dementia: from homologous pathology to region-specific cognitive phenotypes

### The ALS–FTD spectrum: pathological homology and anatomical selectivity

The spectrum encompassing ALS and FTD is characterized by two principal features: shared pathological mechanisms and targeted anatomical impact. Although TDP-43 proteinopathy constitutes a shared molecular foundation, its region-specific distribution gives rise to a heterogeneous clinical phenotype. A significant mechanism may involve the transition from a liquid state to a solid state, influenced by alterations in stress granule dynamics. Cd acts as a potent stressor that prompts TDP-43 to shift from a reversible liquid form to irreversible solid aggregates by stimulating an excess concentration and oxidative mixing within stress granules (Yan et al., 2024).

This region-specific vulnerability may arise from the differential expression of BBB transporters, such as ZIP8, and variations in neuronal metallothionein systems (Ravasia and Hirsch-Reinshagen, 2025; Deneubourg et al., 2022), resulting in enhanced Cd uptake and retention in the frontotemporal cortex relative to the motor cortex (McCabe and Zhao, 2024). Although this hypothesis provides a plausible explanation for phenotypic heterogeneity, high-resolution mapping of metal transport dynamics across distinct regions of the human brain remains limited. Future studies may leverage spatial transcriptomics to accurately assess the three-dimensional relationship between transporter localization and regional cognitive decline within human samples.

### Mechanisms of progression: endo-lysosomal blockage and proteotoxic effects

The progression of FTD associated with Cd is primarily driven by impairment of the endo-lysosomal degradation pathway. Cd disrupts lysosomal acidification, leading to reduced activity of hydrolases, and interferes with the transport and maturation of damaged endosomes into lysosomes, thereby inhibiting the clearance of toxic TDP-43 aggregates (Todd et al., 2023). With this pathway compromised, dysfunction of the adapter protein p62 further exacerbates proteotoxic stress, ultimately leading to the breakdown of neuronal homeostasis (Davidson et al., 2022).

In light of this mechanism, future therapeutic approaches could adopt a multifaceted strategy that includes restoring lysosomal acidification, normalizing endosomal transport, and modulating p62 function to address the limitations of therapies targeting a single pathway.

### Neuroinflammation and social-cognitive impairments

Cd-related cognitive decline extends beyond direct neuronal injury as it also induces aging-like inflammatory responses in microglia through disruption of the immune regulatory factor TBK1, leading to social and cognitive impairments without affecting motor functions. This process closely resembles the early manifestations of social disconnection and reduced empathy observed in patients with frontotemporal dementia (Lenoel et al., 2025), suggesting that Cd may directly affect higher-order social-cognitive brain regions by accelerating “central immune aging” (DeLan et al., 2025).

Current evidence has yet to determine whether microglial aging is a primary toxic effect of Cd or a secondary response to neuronal damage. Future research may employ single-cell sequencing approaches to delineate the temporal sequence of toxic effects across different brain cell populations, thereby identifying the primary immune trigger and informing early intervention strategies in frontotemporal dementia.

### Early-life exposure and developmental neurotoxicity

Cd exerts significant neurotoxic effects on brain development both prenatally and postnatally. Although the placenta serves as a partial barrier to heavy metal exposure, Cd can still traverse this barrier via divalent metal transporters and accumulate at high concentrations within the placental tissue, posing risks to the developing fetus (Zhou et al., 2025a). During this period, the BBB is not yet fully mature, allowing Cd to enter the central nervous system, where it interferes with the proliferation and differentiation of neural stem cells (Preety et al., 2025).

Recent studies using brain organoids have demonstrated that Cd exposure disrupts the formation of primary cilia in neural precursor cells, thereby impairing key developmental signaling pathways such as Wnt and Sonic hedgehog (Shh). These disruptions can lead to significant abnormalities in cortical development (Huang et al., 2021).

There is a well-established association between prenatal Cd exposure and cognitive impairments in children (Kou et al., 2025). However, the relationship between Cd exposure and the risk of autism spectrum disorder (ASD) and attention-deficit/hyperactivity disorder (ADHD) remains less clear. Although placental Cd accumulation has been associated with an increased likelihood of ASD- and ADHD-like symptoms in toddlers approximately 3 years of age, these adverse effects may be modulated by supportive social environments during early childhood (Zhou et al., 2025b).

This complex interplay among environmental, biological, and social factors underscores the importance of a balanced perspective when evaluating the developmental toxicity of Cd.

### Potential interventions and therapeutic strategies: from molecular defense to regenerative repair

**FIGURE 3:**
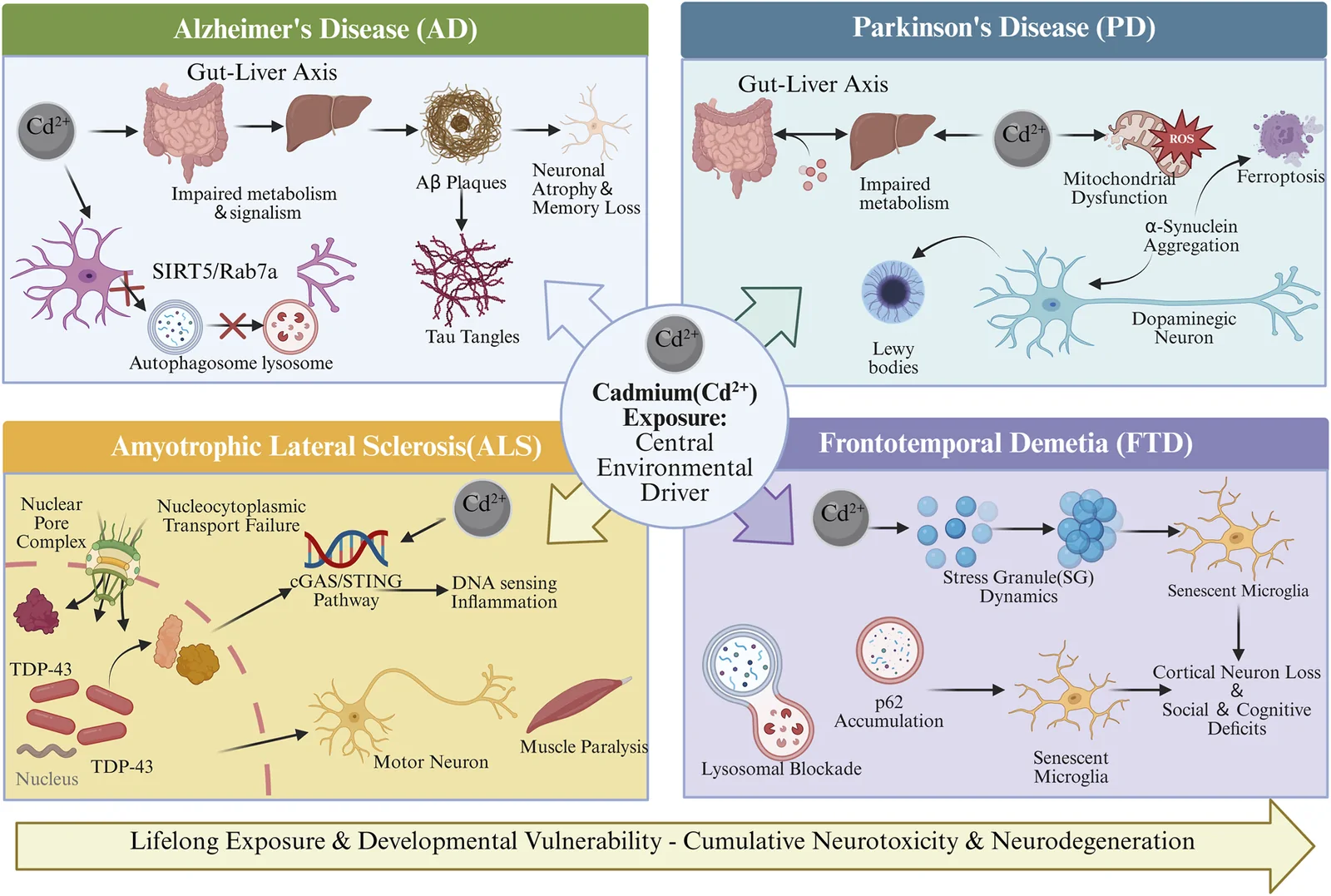
Integrated model of Cd-driven neurodegenerative disorder spectrum through systemic proteotoxicity and gut–liver–brain axis dysfunction.

In response to the complex effects of Cd-induced neurotoxicity, contemporary therapeutic strategies are shifting from a sole reliance on antioxidant approaches toward restoring organelle homeostasis and promoting endogenous repair mechanisms. These interventions are organized around the molecular pathways defined in the preceding sections, including oxidative stress and ferroptosis, mitochondrial dysfunction and autophagic flux, neuroinflammation, calcium dyshomeostasis, and synaptic dysfunction, rather than as a generic list. For each pathway, progress on the proposed targets, translational feasibility, and the evidence gaps that limit clinical application are considered in turn. The framework prioritizes these interventions hierarchically, from neural stem cell regeneration at the highest level to synaptic restoration at the lowest, as depicted in Figure 3. Table 1 summarizes the major strategies, spanning systemic detoxification, drug repurposing, phytochemicals, nutritional interventions, and emerging molecular designs.

In the context of proteostasis, earlier studies have largely relied on antioxidants such as gallic acid to mitigate oxidative stress; however, this approach does not address the intracellular accumulation of misfolded proteins. Conversely, puerarin has been shown to attenuate excessive activation of the mitochondrial unfolded protein response (UPRmt) (Adebiyi et al., 2022); nevertheless, caution is warranted as excessive inhibition may compromise the cell’s intrinsic stress adaptation capacity.

With respect to autophagy regulation, both linagliptin (Arab et al., 2023a) and topiramate (Arab et al., 2023b) enhance the expressions of autophagy-related genes via the SIRT1 signaling pathway, suggesting that therapeutic efficacy lies in restoring the flow of autophagy rather than merely initiating autophagy.

Recent studies delineate five hierarchical therapeutic tiers: beginning with neural stem cell regeneration at the highest level, progressing through immune microenvironment remodeling and maintenance of organelle quality at intermediate levels, and culminating in the restoration of synaptic function at the lowest level. This integrated framework reflects a paradigm shift from “single-target antioxidant strategies” toward “system-level network remodeling,” thereby minimizing redundant investigative efforts, as summarized in Table 1.

Nevertheless, the restoration of synaptic structure and function does not occur synchronously. Although TSG enhances dendritic spine density via the BDNF/TrkB signaling pathway (Gu et al., 2025), structural recovery does not necessarily equate to functional restoration. At the functional level, quercetin primarily upregulates NMDA receptor expression, thereby facilitating long-term potentiation (LTP) (Srivastava et al., 2023), whereas camphor predominantly acts through inhibition of acetylcholinesterase activity (Hamdollahi et al., 2025).

The mechanistic divergence among these interventions suggests that monotherapy is unlikely to fully address complex cognitive deficits, underscoring the necessity for future research into multitarget synergistic strategies.

The therapeutic scope is increasingly expanding toward microenvironmental remodeling. Edaravone inhibits glial proliferation via modulation of the Notch1 signaling pathway (Fan et al., 2021), whereas quetiapine (Althagafy et al., 2024) and curcumin (Namgyal et al., 2021) primarily target the NLRP3 inflammasome to attenuate neuroinflammatory and anxiety-related responses. It is important to note that, given the critical role of Notch1 in maintaining neurogenesis, prolonged inhibition of this pathway may pose significant risks.

At the regenerative level, Wnt3a effectively counteracts the senescence of hippocampal neural stem cells by activating the Wnt/β-catenin signaling pathway (Zhang Y. et al., 2025).

However, most current studies are based on pretreatment or co-treatment paradigms; their efficacy in reversing established pathology remains to be validated through rigorous post-treatment investigations.

From a therapeutic perspective, stabilization of cellular organelles and neuronal regeneration represent key components of recovery, whereas the initial removal of Cd ions remains essential. Nevertheless, clinical management of Cd-induced neurotoxicity faces substantial pharmacokinetic challenges. Conventional hydrophilic chelating agents, such as CaNa_2_EDTA and DMSA, exhibit limited permeability across the BBB, thereby restricting their ability to eliminate accumulated Cd within the brain. Furthermore, these agents may inadvertently redistribute Cd to the brain or induce nonspecific depletion of essential trace elements (Cheng et al., 2025).

Recent evidence suggests that the neuroprotective effects of EDTA may be mediated primarily through systemic anti-inflammatory mechanisms rather than direct chelation within the brain, indicating that the efficacy of hydrophilic chelators in the brain may be less robust than previously assumed.

Given the limitations associated with monotherapeutic approaches, an integrated strategy incorporating lipophilic chelators in combination with antioxidants and zinc supplementation may represent a more effective approach to mitigating tissue oxidative damage (Tandon et al., 2003).

To mitigate the risks associated with direct central nervous system-targeted interventions, recent strategies have focused on limiting Cd entry via the gut–liver–brain axis. Engineered bacterial systems expressing surface-displayed metallothionein have been developed to sequester Cd within the intestinal lumen, thereby interrupting its systemic absorption and subsequent distribution to the liver and brain, ultimately reducing neurotoxicity and hepatotoxicity (Zhang Y. et al., 2024).

In parallel, studies have demonstrated that specific *Lactobacillus* strains can attenuate neuroinflammatory triggers by reinforcing intestinal barrier integrity and limiting endotoxin translocation, thereby offering a promising non-invasive strategy for both the prevention and management of Cd-induced toxicity (Yadav et al., 2025).

### Challenges, assessment, and future perspectives

The integration of human brain organoids with high-density microelectrode arrays provides critical insights into Cd-induced neurotoxicity from both structural and functional perspectives (Carstens et al., 2025; Hu et al., 2025). Nevertheless, a substantial gap persists between *in vitro* models and real-world exposure scenarios. This limitation is particularly evident in the following aspects.

A more basic limitation lies in the exposure paradigms themselves. Most mechanistic data are derived from acute high-dose models, whereas real-world exposure is chronic and occurs at low levels. Even below acute toxicological thresholds, chronic Cd exposure produces neurobehavioral and epigenetic changes in animals at blood levels comparable to those of the general human population (Deng et al., 2023). Such studies, however, typically evaluate single doses, leaving the activation thresholds and cumulative epigenetic effects of low-dose exposure largely unexplored. Real-world exposure also involves pollutant mixtures, which generate DNA methylation patterns distinct from those observed in single-exposure studies (Chen et al., 2024). Vulnerable groups, such as pregnant women, children, and people with metabolic disease, show heightened low-dose susceptibility yet remain underrepresented in mechanistic work (Zhu et al., 2025; Gonzalez-Villalva et al., 2026). Direct pathological comparison of high- and low-dose regimens, together with systematic mapping of dose-dependent signaling thresholds, remains a critical gap that must be closed before experimental findings can inform public health risk assessment.

First, the complex pharmacokinetic processes governing transport of Cd across the BBB and its systemic trafficking along the gut–liver–brain axis, as discussed previously, are not recapitulated in current organoid systems. Direct exposure via culture media bypasses key physiological mechanisms, including efflux transporters such as multidrug resistance-associated proteins (MRPs), thereby introducing significant uncertainty in extrapolating experimental findings on “low-dose” exposure to “safe limits” for human health.

Moreover, Cd can persist in the renal cortex for 10–30 years, whereas the short culture windows of organoid systems cannot reproduce such long-term epigenetic effects. Reliance on a single induced pluripotent stem cell (iPSC) line further fails to capture the genetic variability of key transporters such as ZIP8 and ZIP14 or of associated regulatory genes (e.g., MT1A and ABCB1), potentially masking susceptibility in vulnerable populations (Hermann et al., 2021; Li et al., 2018; Smart et al., 2024).

Furthermore, real-world Cd exposure typically involves complex mixtures of environmental pollutants, which contrasts markedly with the single-toxin paradigms employed in most *in vitro* studies.

Consequently, a major challenge moving forward is the development of advanced microphysiological systems incorporating multi-organ-on-a-chip (MOC) platforms to more accurately model systemic pharmacokinetics. Simultaneously, it is essential to establish quantitative correlations between *in vitro* electrophysiological abnormalities and clinically relevant biomarkers. Such an approach would facilitate the translation of experimental findings into actionable public health strategies for identifying high-risk populations and enabling timely interventions.

## Conclusion

The neurotoxic profile of Cd extends beyond any single biochemical pathway. The depletion of GSH, induction of ferroptosis, calcium dyshomeostasis, and mitochondrial dysfunction are not isolated events; rather, they converge into a self-reinforcing positive feedback loop. This synergistic cascade provides a mechanistic basis for the persistent neurological impairment associated with Cd exposure, even at sub-toxic, chronic levels. Furthermore, the pathological convergence observed across the spectrum of AD, PD, and the ALS–FTD suggests that Cd-induced neurodegeneration represents a systemic disruption of proteostasis networks.

From a therapeutic standpoint, conventional hydrophilic chelation remains limited by the BBB and the risk of peripheral-to-central redistribution of Cd, necessitating a transition toward more advanced delivery strategies. Interventions targeting the gut–liver–brain axis, such as the *in situ* adsorption via EcN-MT-engineered bacteria, represent a promising alternative; however, their clinical colonization dynamics require further validation. Ultimately, bridging the gap between experimental research and public health applications remains a critical priority. Translating *in vitro* findings, such as NCOA4-mediated ferroptotic signaling, into quantifiable, blood-based biomarkers will require large-scale prospective cohort studies integrated with MOC platforms to enable precision-targeted interventions in at-risk populations.

## Glossary

Aβ: Amyloid-beta
AChE: Acetylcholinesterase
AD: Alzheimer’s disease
ALS: Amyotrophic lateral sclerosis
ApoE4: Apolipoprotein E4
APP: Amyloid precursor protein
BACE1: Beta-site APP-cleaving enzyme 1
BBB: Blood–brain barrier
BDNF: Brain-derived neurotrophic factor
Cd: Cadmium
cGAS: Cyclic GMP–AMP synthase
DAMP: Damage-associated molecular pattern
DSST: Digit symbol substitution test
EDTA: Ethylenediaminetetraacetic acid
ERAD: Endoplasmic reticulum-associated degradation
ETC: Electron transport chain
FTD: Frontotemporal dementia
GABA: Gamma-aminobutyric acid
GPx4: Glutathione peroxidase 4
GSH: Glutathione
GSK-3β: Glycogen synthase kinase-3 beta
IL-1β: Interleukin-1 beta
iPSC: Induced pluripotent stem cell
MAPK: Mitogen-activated protein kinase
MCI: Mild cognitive impairment
MDA: Malondialdehyde
mPTP: Mitochondrial permeability transition pore
MT: Metallothionein
mtDNA: Mitochondrial DNA
mtROS: Mitochondrial reactive oxygen species
NCOA4: Nuclear receptor coactivator 4
NF-κB: Nuclear factor kappa B
NFTs: Neurofibrillary tangles
NHANES: National Health and Nutrition Examination Survey
NLRP3: NLR family pyrin domain-containing 3
NMDA: N-methyl-D-aspartate
PD: Parkinson’s disease
PS1: Presenilin-1
ROS: Reactive oxygen species
SCFAs: Short-chain fatty acids
SIRT: Sirtuin
SOD: Superoxide dismutase
STING: Stimulator of interferon genes
TDP-43: TAR DNA-binding protein 43
TNF-α: Tumor necrosis factor-alpha
TUDCA: Tauroursodeoxycholic acid
α-Syn: Alpha-synuclein
